# Facile Synthesis of Silver Nanoparticles From Sustainable Sargassum sp. Seaweed Material and Its Anti-inflammatory Application

**DOI:** 10.7759/cureus.57754

**Published:** 2024-04-07

**Authors:** Kavishri S, Geetha A, Ilangovar I G K, Vasugi S, Sivaperumal P, Balachandran S

**Affiliations:** 1 Department of Physiology, Saveetha Dental College and Hospitals, Saveetha Institute of Medical and Technical Science, Saveetha University, Chennai, IND; 2 Department of Prosthodontics, Saveetha Dental College and Hospitals, Saveetha Institute of Medical and Technical Science, Saveetha University, Chennai, IND

**Keywords:** anti-inflammatory behavior, xrd analysis, highly crystalline, sargassum sp. seaweed, silver nanoparticles

## Abstract

Background

Sustainable and environmentally friendly methods of producing nanoparticles are now being investigated by scientists. Because there are so many marine renewable resources, scientists are focusing their attention on studying seagrass, seaweed, mangroves, marine macroalgae, and microalgae. An exciting new frontier in research involves the synthesis of nanoparticles using extracts from seaweed. Seaweed extracts are utilized to synthesize silver nanoparticles (Ag NPs), which serve as both reducing and stabilizing agents. Seaweed extracts possess bioactive substances like proteins, polysaccharides, and polyphenols that enable them to effectively convert silver (Ag^+^) ions into Ag NPs. Ag NPs derived from *Sargassum* seaweed have played an essential role in improving the anti-inflammatory properties of seaweed extracts. This study aimed to investigate the biosynthesis of Ag NPs from *Sargassum* seaweed and evaluate their anti-inflammatory properties.

Materials and methods

About 50 g of seaweed samples were mixed with 100 mL of distilled water and stirred for 24 hours. Additionally, 1.2 g of silver nitrate (0.120 M) was dissolved in 60 mL of distilled water to make a silver (Ag) solution. A 60 mL solution of silver nitrate (AgNO_3_) was mixed with a 40 mL solution of seaweed extract in water, and the mixture was stirred with a stirrer for 24 hours. A UV spectrophotometer was used to regularly monitor the reduction of Ag^+^ ions in the solution. Ag NPs were purified using a sequence of centrifugation steps with a duration of 10 minutes at a speed of 2500 revolutions per minute (rpm). To remove moisture from the water-suspended nanoparticles, they were vacuum-dried for 24 hours.

Results

The synthesis of Ag NPs from seaweed extract resulted in a noticeable change in the color of the mixture, which went from pale to brown. The alteration in color signifies the reduction of AgNO_3_ to Ag^+^ ions, facilitating the creation of Ag NPs. X-ray diffraction (XRD) measurement verified the remarkable crystallinity of the synthesized Ag NPs. Field emission scanning electron microscopy (FESEM) images indicated a spherical, homogeneous structure. The Ag NPs derived from seaweed exhibited significant anti-inflammatory characteristics.

Conclusion

Utilizing *Sargassum* sp. seaweed in the biological synthesis of Ag NPs shows promise to develop nanomaterials that can exhibit anti-inflammatory effects. This technique has benefits, such as being environmentally friendly and cost-efficient. Additional research in this area is essential for effectively exploiting the potential of Ag NPs in anti-inflammatory activity.

## Introduction

Nanoparticles can be synthesized through various chemical and physical techniques, including the sol-gel process [1], co-precipitation method [2], thermal decomposition [3], laser ablation [4], and spray pyrolysis [5]. Although numerous chemical techniques exist for synthesizing metal nanoparticles, these reactions often involve toxic and hazardous reactants and starting materials. This problem could potentially be resolved by increasing the production of biocompatible nanoparticles through the process of biological synthesis, utilizing plants and marine resources. Environmental concerns about chemical synthesis routes have led to attempts at biological synthesis [6,7].

The biological synthesis of metal nanoparticles has garnered significant attention due to their enhanced environmentally friendly characteristics in material synthesis. Biologically synthesized nanoparticles offer superior control over the size distribution compared to alternative methods. Because of its many benefits over traditional chemical approaches, nanoparticle production using biological processes, such as microbes, biomolecules, and plant extracts, has become an attractive strategy. According to many studies, biologically produced nanoparticles have better control over size distribution than other approaches. As an example, research done by Ahmad showed that, in contrast to chemically manufactured gold nanoparticles, those made utilizing plant extracts had a more uniform shape and a narrower size dispersion. Mittal found that biomolecules in plant extract controlled the nucleation and development of nanoparticles by acting as stabilizing agents and reducing agents. The study by Iravani also found that instead of nanoparticles made chemically, those made utilizing microbial systems had a more uniform size distribution and were more monodisperse. The enzymatic activity and biomolecules found in the microbial culture allowed for the controlled synthesis of nanoparticles with a variety of sizes and shapes via the biological synthesis process. In addition, Mittal emphasized the benefits of biologically produced nanoparticles in their review, highlighting their scalability, ecological friendliness, and repeatability. Nanoparticles with a more consistent size distribution and improved stability are produced by precisely controlling the reaction conditions, which is made possible by the intrinsic features of biological systems [8-11]. The noble metals silver (Ag), copper (Cu), platinum (Pt), gold (Au), and palladium (Pd) are frequently employed for the synthesis of metallic nanoparticles. Ag is the favored noble metal in the domains of biological systems, living organisms, and medicine [12,13]. Research has been conducted on the anti-inflammatory properties of silver nanoparticles (Ag NPs). Through the regulation of immune responses and the inhibition of inflammatory pathways, they possess potential anti-inflammatory properties. The specialized anti-inflammatory action of Ag NPs is facilitated by their small size and high surface area, which enhances their interaction with cells [14,15].

*Sargassum* sp. seaweed extract is becoming recognized in biomedicine for its abundant bioactive components that may have therapeutic benefits. *Sargassum* sp. seaweed is recognized for its wide range of biomolecules, which include different chemicals that may have health-promoting properties. The composition may vary according to the* Sargassum* species and ambient conditions. Polyphenols are a category of plant chemicals known for their antioxidant characteristics. Fucoidans are intricate sulfated polysaccharides found in brown seaweeds, such as *Sargassum*. Alginates are a form of polysaccharide present in *Sargassum* seaweed, and they contain sterols crucial for cell membrane structure. Certain sterols may reduce cholesterol levels. The distinctive nutritional and bioactive profile of *Sargassum* seaweed is a result of the combination of these substances [16].

Researchers are studying the possible health advantages of these substances, with a focus on examining the uses of *Sargassum* seaweed in functional meals, nutraceuticals, and medications. *Sargassum* extracts are recognized for their antioxidants that help counteract detrimental free radicals in the body. Antioxidants are essential in reducing oxidative stress, which is linked to many chronic illnesses such as cancer and cardiovascular ailments. The compounds found in *Sargassum* seaweed extract have anti-inflammatory effects. This putative anti-inflammatory effect may be helpful in treating inflammatory disorders and illnesses. *Sargassum* seaweed extracts have antibacterial activity against a variety of bacteria, fungi, and viruses. This indicates a possible function in creating antimicrobial substances to treat infections and enhance general immunological health [17].

Research has investigated the anti-cancer effects of *Sargassum* seaweed extracts. The bioactive chemicals may have cytotoxic effects on cancer cells and disrupt mechanisms linked to cancer growth and advancement. Research suggests that *Sargassum* seaweed extracts may possess anti-diabetic properties by impacting glucose metabolism and insulin sensitivity. This makes them a focal point in diabetes studies. Researchers are now extracting and analyzing certain bioactive chemicals found in *Sargassum* extracts to better understand their modes of action and possible uses in medication development. *Sargassum *seaweed is a nutrient-rich food that contains carotenoids, cellulase, and protein, as well as aspartic and glutamic acids. The polysaccharides present in *Sargassum* seaweed help maintain healthy levels of blood sugar and blood pressure.

Seaweed, which encompasses various types of algae such as green, brown, and red, exhibits a diverse array of biological activities, including cytotoxic, anti-inflammatory, antibacterial, and antiviral properties. Seaweeds are widely regarded as the most promising reservoirs of bioactive primary and secondary metabolites. They have been employed in food products, wastewater treatment, and pharmaceutical applications. Seaweed contains phytochemicals with hydroxyl, carboxyl, and amino functional groups. These compounds can be utilized as capping agents to form a robust coating on metal nanoparticles. Additionally, they serve as effective agents for reducing metals [18-22]. The stable, non-toxic, and biologically active properties of *Sargassum *species make them suitable for use as a bio-source in the synthesis of nanoparticles.

The toxicity of nanoparticles is greatly influenced by their size and surface features. Because small nanoparticles have a greater surface area per unit mass, they are more likely to be reactive and potentially dangerous. Their interactions with biological systems may also be influenced by surface coatings or functionalization. Ag NPs produced biologically have the ability to release Ag^+^ ions into the environment. The cytotoxicity of Ag NPs produced biologically is often lower than that of Ag NPs produced chemically; nevertheless, the safety profiles of these two types of nanoparticles might differ according to characteristics including size, shape, surface charge, and surface functionalization. This study presents a fast technique for producing Ag NPs under normal conditions by utilizing an extract solution derived from *Sargassum* seaweed, a type of brown seaweed known for its potential anti-inflammatory properties. Notably, this method does not require the use of accelerators, template-shaping agents, or additives to prevent particle aggregation. It offers a high production of nanomaterials, accompanied by exceptional optical characteristics. The process is elucidated through various analytical methodologies, such as X-ray diffraction analysis (XRD), field emission scanning electron microscopy (FESEM), transmission electron microscopy (TEM), and its anti-inflammatory activities. 

## Materials and methods

Origin of the sample and preparation of seaweed extract

The seaweed specimens used for this research, *Sargassum *sp., were from the Tuticorin area in Tamil Nadu, India. The seaweed was washed extensively with normal water to eliminate any floating debris. Temperature was set below 60℃ for the drying of seaweed materials. After the drying process, the seaweed was ground into granules, and then it underwent the distillation process at 80℃ using ethanol as a solvent. 

In the extraction process, it is necessary to filter the mixture to eliminate solid residues, resulting in a transparent extract. Evaporation is a method that can accomplish these objectives effectively. The extract of *Sargassum *sp. seaweed was properly stored to ensure its stability and quality. Typically, the process entails preserving the extract in airtight containers, shielded from light and heat, to avoid deterioration. We added this solution dropwise to Ag precursors for the synthesis of Ag NPs, acting as a reducing and stabilizing agent. As reducing agents, the bioactive compounds in seaweed extract will lower the concentration of Ag^+^ ions in nanoparticles.

Synthesis of silver nanoparticles (Ag NPs)

A solution of silver precursor was combined with an extract of *Sargassum* sp. seaweed in a controlled environment, including temperature and pH (8). About 1.2 g of silver nitrate (AgNO_3_) (0.120 M) was introduced into a conical flask containing 60 mL of distilled water. The resultant mixture was subsequently agitated for approximately 24 hours at 70°C, cooled, and the extract was then filtered through a Whatman filter paper (Whatman Plc, Maidstone, UK). A 40 mL (5 mg/mL) aqueous seaweed extract was slowly introduced into a 60 mL solution of AgNO_3_. The mixture was continuously stirred for 24 hours at a speed of 580 rpm using a stirrer at a temperature of 70°C and the pH of the solution was 7. The concentration of Ag^+^ ions in the solution was periodically monitored using a UV spectrophotometer. The Ag NPs were purified using a series of centrifugation procedures, each of which proceeded at a speed of 2500 rpm for 10 minutes. The water-suspended nanoparticles underwent a 24-hour vacuum drying process to remove moisture from the nanoparticles. The dried sample was later tested for anti-inflammatory properties [19]. To reduce Ag^+^ ions to elemental silver (Ag⁰), the phytochemicals in the seaweed extract work as reducing agents. Ag NPs form and expand in the solution as a result of this reduction process.

Material characterization

Ag NPs were analyzed using XRD patterns on a Rigaku D/Max-2000 diffractometer (Rigaku Corporation, Japan) with Cu Kα radiation (λ = 0.15406 nm) to determine their phase. Data was recorded in a 2θ range of 10-80° at 0.05°/s. A field-emission scanning electron microscope (FESEM, FEI, Quanta 200F) was used to examine the Ag NP sizes, surface morphology, and structural porosity after a 180-second coating with platinum. The shutter speed was set at 5 mm with an acceleration voltage of 15 kV. The images were taken using a JEM-2100F electron microscope (JEOL Ltd., Akishima, Japan) with an accelerating voltage of 200 kV and included TEM, high-resolution transmission electron microscopy (HRTEM). Three drops of the sample ethanol solution were used to create the TEM samples, which were then dried in the environment on a copper grid covered with carbon film.

Anti-inflammatory activity

A protein denaturation experiment was carried out as per the methods described by Gupta, with a few improvements noted by Gunathilake. The anti-inflammatory efficacy of the Ag NPs extract was assessed at different concentrations by dissolving it in a dimethyl sulfoxide (DMSO) solution. After the incubation and heating procedures, the measurement of absorbance at a specific wavelength of 660 nm was conducted. The inhibition percentage was calculated by comparing the effects of diclofenac sodium, a reference standard drug, with those of DMSO, the control. An amount ranging from 25 to 125 μg of the Ag NPs was added to 0.2 mL of 1% bovine albumin, 4.78 mL of phosphate-buffered saline (PBS, pH 6.3), and 5 mL of the reaction mixture. Everything was combined and placed in a water bath set at 37°C for 15 minutes. After that, it was heated to 70°C for 5 minutes. Using the Optima SP-3000 UV/VIS spectrometer (Optima, Japan) the turbidity was measured at 660 nm during the cooling process. The protein's denaturation inhibition percentage was calculated using the procedure below. To get the percentage inhibition of denaturation, 100 was multiplied by (1 - A2/A1), where A1 is the absorption of the control sample and A2 is the absorption of the test sample.

## Results

X-ray diffraction (XRD)

**Figure 1:**
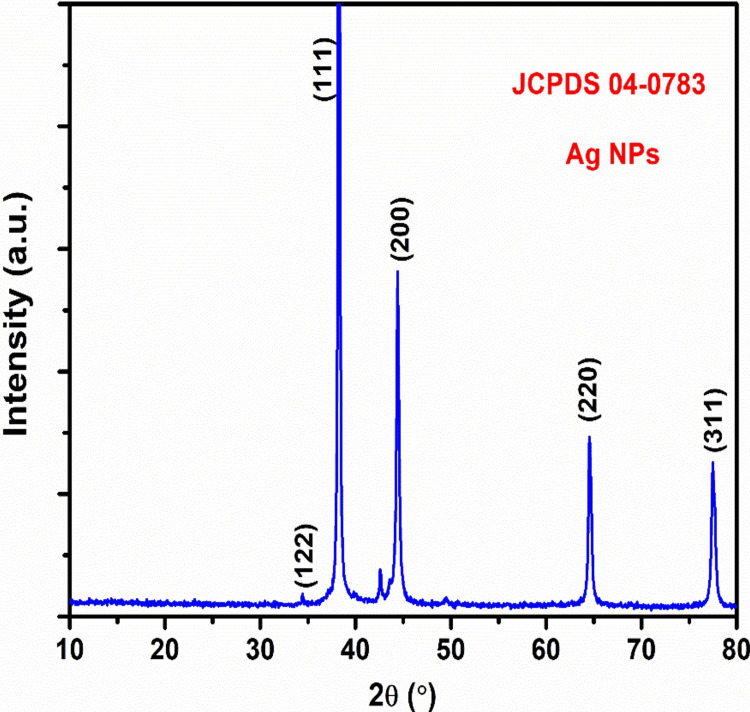
XRD analysis of Ag NPs using Sargassum sp. seaweed XRD: X-ray diffraction; Ag NPs: Silver nanoparticles; JCPDS: Joint Committee on Powder Diffraction Standards

XRD analysis is a rapid analytical method mainly employed for identifying the phases of a crystalline material and determining the dimensions of the unit cell. It was confirmed that the XRD pattern of the biosynthesized Ag NPs was correct when a clear peak was found in the analysis. XRD was used to check the crystallinity of the Ag NPs that were made with *Sargassum *sp. seaweed extract. Four prominent diffraction peaks were identified in the XRD pattern at 2θ = 34.02°, 37.80°, 44.20°, 64.41°, and 77.09°. XRD patterns showed clear peaks, which matched the crystallographic planes (122), (111), (200), (220), and (311) that are unique to Ag NPs. The diffraction peaks (111), (200), and (220) confirmed the face-centered cubic (fcc) structure of Ag, with the sharpness of these peaks indicating the presence of high crystallinity of nanosized particles. The highly intense peaks at (111) indicated good crystallinity of the Ag NPs. The diffraction pattern matched the standard, Joint Committee on Powder Diffraction Standards (JCPDS) No. 04-0783. The analysis revealed no spurious diffractions due to crystallographic impurities. The obtained XRD pattern was consistent with the early reported documentation of Ag NPs synthesized using *Phyllanthus maderaspatensis* L. root extract. The strong peak and wide diffraction pattern indicated that the synthesized system has a nanodimensional state (Figure 1).

Field emission scanning electron microscopy (FESEM)

**Figure 2:**
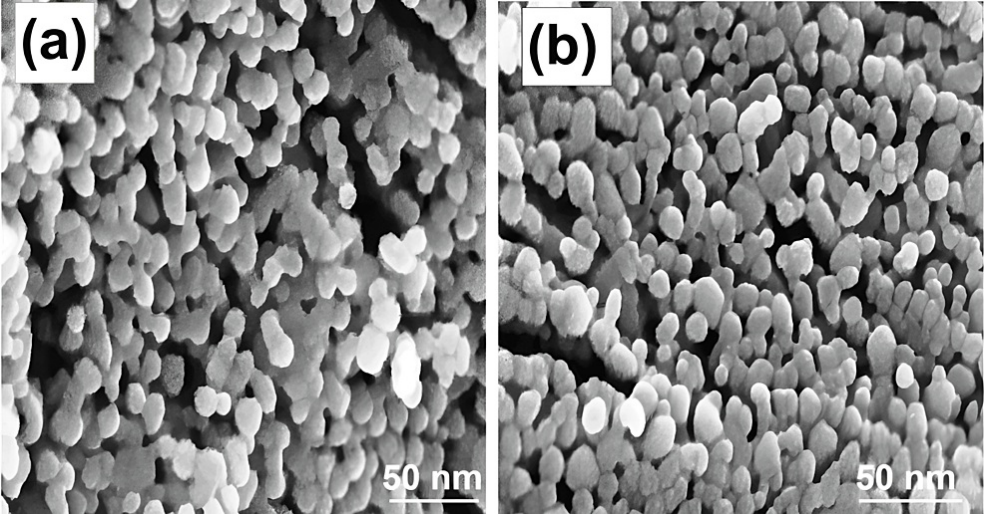
FESEM analysis of Ag NPs using Sargassum sp. seaweed FESEM: Field emission scanning electron microscopy; Ag NPs: Silver nanoparticles

Metal nanoparticles such as silver and gold have great electrical conductivity, making them suitable for studies using FESEM. The morphology and size of the synthesized Ag NPs using *Sargassum* sp. seaweed extract were examined through FESEM. The FESEM image revealed the spherical morphology of Ag NPs. The FESEM images showed that the Ag NPs exhibited an agglomerated structure due to the drying process, indicating that nanoparticles were mostly uniform with a narrow size distribution (Figure 2). The Ag NP surface exhibited a high degree of porosity, with many voids present. This feature greatly promoted surface catalytic reactions. Their uniform spherical shape makes it easy to measure them precisely, which helps us understand how they behave and interact with one another. The high-resolution images also showed how their stability and responsiveness are affected by their dispersion and aggregation tendencies. Optimal synthesis techniques and property tailoring for varied biological, environmental, and technological applications may be informed by understanding the structural subtleties of spherical Ag NPs by FESEM.

Transmission electron microscopy (TEM)

**Figure 3:**
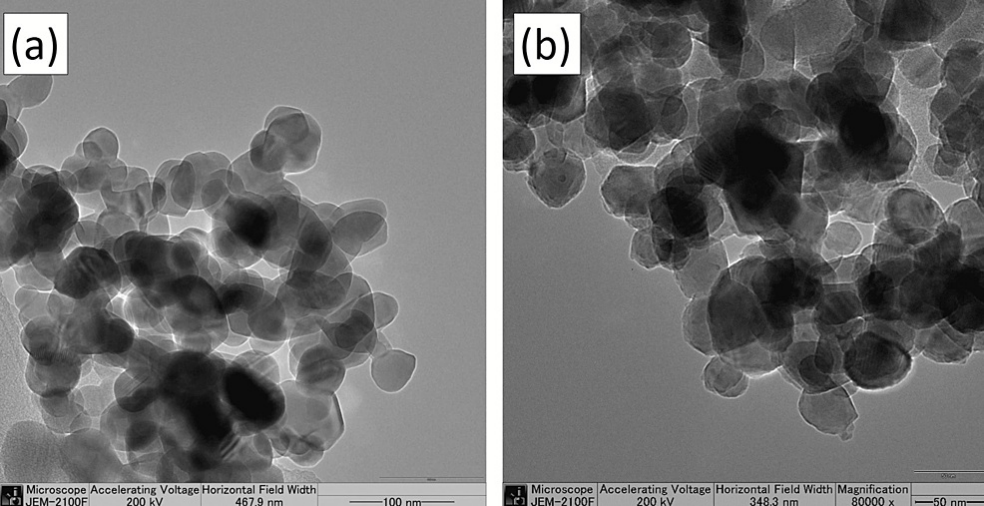
TEM analysis of Ag NPs using Sargassum sp. seaweed at (a) 100 nm and (b) 50 nm TEM: Transmission electron microscopy; Ag NPs: Silver nanoparticles

TEM images of Ag NPs were recorded on a JEM-2100F electron microscope operated at 200 kV. TEM offers accurate and high-resolution imaging of nanoparticles at the nanometer scale, providing detailed information about their size, shape, morphology, aggregation state, and distribution. This technique involves using a beam of electrons to interact with the ultrathin sample. It helps to understand the function of capping agents and Ag NPs metabolite encapsulation. TEM offers the best resolution for discovering atomic packing in nanoparticles, allowing for the detection of crystal imperfections. The analysis of the high-resolution TEM image confirmed that the form of the nanoparticles is mostly spherical in shape. There was some variation in form and size, primarily caused by weaker interactions. The TEM revealed that the Ag NPs were of varying sizes and were surrounded by a thin layer, which could be organic compounds (stabilizing agents) in the *Sargassum* sp. seaweed (Figure 3).

Anti-inflammatory activity of Ag NPs derived from seaweed

**Figure 4:**
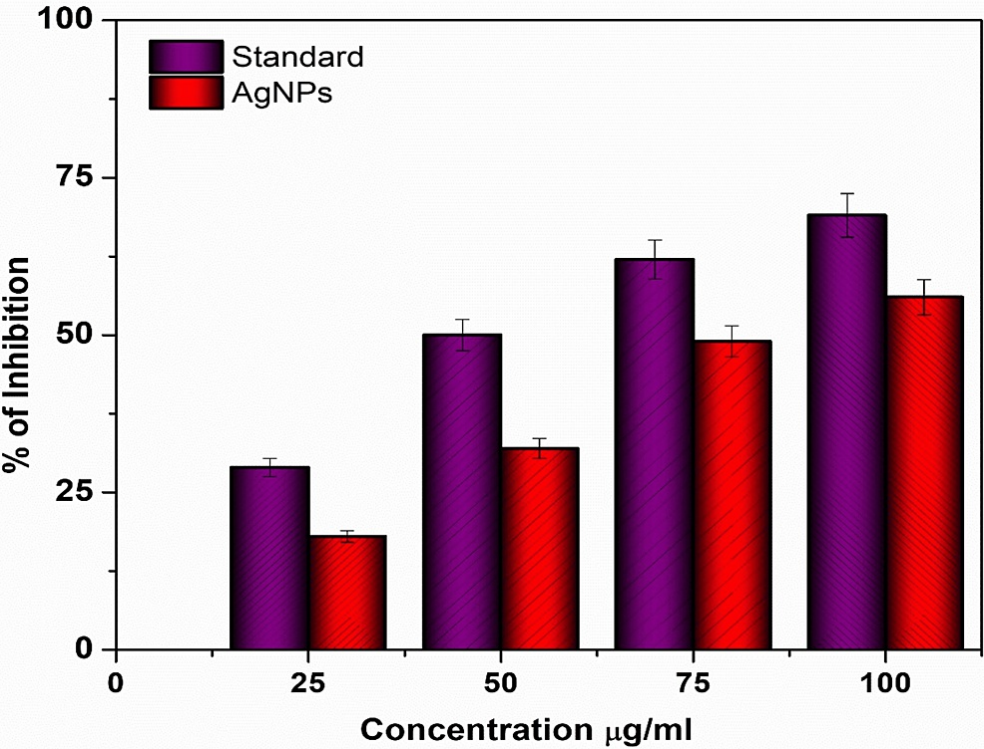
Anti-inflammatory activity of Ag NPs from Sargassum sp. seaweed at different concentrations Ag NPs: Silver nanoparticles

Figure 4 illustrates the anti-inflammatory effect of Ag NPs with seaweed extract at different concentrations in comparison to standard values. The protein denaturation activity of diclofenac sodium, which is the standard medication, was found to be 69.2%. The Ag NPs exhibited the highest degree of anti-inflammatory activity, with a score of 58.5%. The concentrations of the sample and the standard were both modified to match a value of 50 µg/mL for the purpose of normalization. The anti-inflammatory effects of Ag NPs made from *Sargassum* sp. seaweed extracts are due to both the specific nanoparticle characteristics and the bioactive chemicals found in the seaweed, especially those involved in green synthesis. Polyphenols, fucoidans, and phlorotannin are only a few of the bioactive components found in *Sargassum *sp. seaweed extract that have anti-inflammatory effects. Ag NPs synthesized from seaweed extract have shown significant medicinal promise in investigations into their anti-inflammatory activities. The capacity of Ag NPs produced from seaweed extracts to reduce inflammation in several experimental contexts attests to their strong anti-inflammatory properties. Seaweed contains bioactive components, and this work sheds light on how these components interact with nanoparticles, specifically in relation to green synthesis. The Ag NPs showed better anti-inflammatory action and moderate than the diclofenac sodium, highlighting their promise as powerful anti-inflammatory medicines.

During the synthesis of Ag NPs, these chemicals might serve as agents that reduce and stabilize. The release of pro-inflammatory cytokines, including interleukins and tumor necrosis factor-alpha (TNF-α), may be influenced by Ag NPs. The inflammatory response may be dampened by Ag NPs because they decrease the generation of these mediators. *Sargassum* sp. seaweed and Ag NPs both contain bioactive chemicals that may have antioxidant effects. Inhibiting oxidative stress and inflammatory enzyme activity, including cyclooxygenase (COX) and lipoxygenase (LOX), is possible via their ability to scavenge free radicals and reactive oxygen species (ROS). The activities of immune cells, especially macrophages, may be affected by interactions with Ag NPs. Modulating macrophage activity may influence immunological and inflammatory processes generally because of their prominent involvement in the inflammatory response. Inflammation also involves neutrophils, another kind of immune cell. The anti-inflammatory effects of neutrophils may be mitigated by Ag NPs, which impact their function. The nuclear factor kappa B (NF-κB) signaling pathway, an important regulator of inflammatory gene expression, might be disrupted by Ag NPs. Reducing transcription of pro-inflammatory genes may be achieved by inhibiting NF-κB. Inflammation-fighting immune cell adhesion and migration may be mitigated by Ag NPs by downregulating adhesion molecules on endothelial cells. There are a number of variables that may influence the anti-inflammatory effects of Ag NPs produced by *Sargassum* sp. seaweed extract. These include the nanoparticles' size, shape, concentration, and surface properties. When thinking about possible biological uses for Ag NPs, it's important to keep their safety and biocompatibility in focus. There is a need to evaluate the concentrations of inflammatory mediators such as prostaglandins, cytokines (TNF-α, IL-6, and IL-1β), and chemokines in inflammation models treated with Ag NPs in experiments and determine the relative efficacy of biologically produced and chemically manufactured Ag NPs in regulating inflammation by comparing the decrease in inflammatory mediator levels.

## Discussion

The biological synthesis process offers a diverse range of environmentally friendly techniques, cost-effective production, and minimal time requirements. Simultaneously, biologically produced metal nanoparticles have numerous applications in the domains of medicine and agriculture. Nanomaterials have garnered significant interest in recent years due to their potential as anti-inflammatory agents. This discussion centers on the comparative analysis of Ag NPs produced from* Sargassum *seaweed extracts, with a particular emphasis on their anti-inflammatory properties. The process of synthesizing Ag NPs using seaweed extracts was conducted. The presence of brown in the reaction vessels indicated the synthesis of Ag NPs.

The purpose of this analysis was to clarify the potential therapeutic benefits of these nanoparticles in reducing inflammation and their appropriateness for different uses. It is important to mention that Ag NPs consistently exhibited a slightly greater level of inhibition, especially at lower concentrations (10 and 20 µg/mL). The findings suggest that Ag NPs may provide a modest advantage in preventing protein denaturation, indicating their potential efficacy in reducing inflammation. Ag NPs demonstrated efficient stabilization of cell membranes, exhibiting no noticeable disparity between them. This suggests that both Ag NPs can effectively stabilize cell membranes, indicating their equal potential in this aspect of anti-inflammatory activity.

Seaweed has been utilized in the past to produce various nanoparticles, including copper nanoparticles (Cu NPs) derived from the *Ulva lactuca* seaweed. The study discovered that an evaluation of the overall antioxidant capacity and ability to scavenge free radicals suggests that incorporating Cu NPs into *Ulva lactuca* has the potential to augment their antioxidant activity. Upon synthesis, Cu NPs exhibited significant antioxidant properties by effectively neutralizing free radicals and providing defense against ROS. The synthesis of Cu NPs from* Ulva lactuca* provides an environmentally conscious and sustainable method for fabricating nanoparticles [23].

A study was conducted to demonstrate the anti-inflammatory activity of Ag NPs that were synthesized using an extract from the leaves of *Cymodocea rotundata*. The study discovered that these Ag NPs demonstrated a level of anti-inflammatory effectiveness similar to that of conventional medications when given at a concentration of 100 μg/mL. This indicates the possible effectiveness of Ag NPs in controlling inflammation. Although Ag NPs exhibit anti-inflammatory properties, they can also induce pro-inflammatory responses in the presence of ROS. The contrasting effects highlight the importance of having a thorough understanding of the mechanisms and contexts in which Ag NPs are used in anti-inflammatory applications [24].

An extensive review delves deeply into the characteristics, production techniques, and several uses of Ag NPs. Examining Ag NPs from a physical, chemical, and biological perspective, the study draws attention to their distinctive properties. Researchers and professionals seeking a comprehensive knowledge of Ag NPs will find this review beneficial. It provides insights into a variety of synthesis processes and applications in materials science and engineering [25]. Another research delves into the uses of Ag NPs in creating a topical antibacterial gel, with a particular emphasis on their medicinal potential. The study examines the antibacterial effectiveness of a gel formulated with Ag NPs and its pharmaceutical formulations to combat microbial infections, bridging the gap between nanotechnology and therapeutics [26]. A study looks into the green production of Ag NPs using an aqueous leaf extract of *Azadirachta indica* (Neem), focusing on environmentally friendly ways to make them. Using plant extracts in the synthesis process upholds the principles of green chemistry. This study highlights sustainable methods for synthesizing Ag NPs by exploring the reducing and stabilizing agent *Azadirachta indica* [27]. Ag NPs biosynthesized in this work target gram-positive and gram-negative bacteria. Research on the synergistic effects of Ag NPs with antibiotics provides insights into possible methods to improve the antibacterial capabilities of already used medications. This study contributes to nanomedicine by investigating potential new ways to fight bacterial infections [28].

Limitations

The presence of contaminants originating from biological sources or reaction conditions can have an impact on both the purity and stability of the synthesized Ag NPs. These changes can negatively impact the quality of the substances and restrict their effectiveness in reducing inflammation. While biological synthesis methods are usually considered scalable, there might be difficulties in ensuring constant reaction conditions and managing factors like pH, temperature, and agitation on a larger scale. These issues can have an impact on the productivity and characteristics of the synthesized Ag NPs. It is important to emphasize that Ag NPs have the potential to act as anti-inflammatory agents, but their effectiveness and appropriateness depend on multiple factors. To effectively utilize nanoparticles in anti-inflammatory therapies, it is crucial to have a thorough understanding of their medicinal properties and safety attributes. At lower concentrations, the nanoparticles exhibit good tolerance, thereby facilitating anti-inflammatory effects. Elevated levels may exceed a critical point at which the harmful effects become more noticeable, leading to cellular strain and triggering the activation of antioxidant defense mechanisms in response to the heightened oxidative load. Recent studies have found that green-synthesized Ag NPs could be effective tools in the fight against inflammation.

## Conclusions

Ag NPs were prepared by a simple, cost-effective method using naturally available *Sargassum Sp.* seaweed, which contains rich bioactive compounds with functional groups that can effectively reduce and stabilize Ag NPs. The prepared Ag NPs were characterized by XRD, SEM, and TEM analysis. The XRD analysis showed the diffraction planes of (111), (200), and (220) that confirm Ag is in the fcc phase. The sharpness of these peaks showed that the Ag NPs are highly crystallized. The SEM images showed that the Ag NPs were crystallized into a spherical shape. The agglomerated shape of the Ag NPs seen in the scanning electron micrographs suggests that their size distribution was quite narrow and homogenous. The high-resolution TEM image revealed that the nanoparticles mostly exhibit a spherical morphology. Some variation in shape and size was seen, mostly due to weaker interactions. The anti-inflammatory behavior of Ag NPs with seaweed extract by the protein denaturation method, as compared to standard values, was boosted by Ag NPs with seaweed extract at varied doses. The usual treatment, diclofenac sodium, has a protein denaturation activity of 69.2%. The anti-inflammatory activity of the Ag NPs was the most significant, obtaining 58.5%.
